# Supplementary material for: Harmonizing sound and light: X-ray imaging unveils acoustic signatures of stochastic inter-regime instabilities during laser melting
Source: Nat Commun. 2023 Dec 5;14:8008. doi: 10.1038/s41467-023-43371-3 (PMC10697982; doi:10.1038/s41467-023-43371-3)
Supplement: Supplementary file 1 — Supplementary Information [file 41467_2023_43371_MOESM1_ESM.pdf]

Supplementary Information for Harmonizing Sound and Light: X-Ray Imaging Unveils Acoustic Signatures of Stochastic Inter-Regime Instabilities during Laser Melting

Supplementary Method 1

Assessment of total energy absorption

The mean keyhole wall angle $\bar{\theta}_w$ for each radiograph was determined using the procedure explained in Kaplan [5], where the front and rear keyhole wall angles are measured between equally distanced points alongside the depth of the keyhole depression zone, followed up by an average of the measured angles. Consequently, the total number of reflections n_{mr} was calculated via $n_{mr} = \pi/4\bar{\theta}_w$, assuming a limiting reflection angle of $\pi/2$, after which there would be no more reflections. Excluding the first reflection, the reflection coefficient for multiple reflections ρ_{mr} can be calculated according to $\rho_{mr} = [\rho_{Fr}(\theta = \pi/2)]^{(n_{mr}-1)}$ where, ρ_{Fr} is the Fresnel reflectivity assuming a normal incident angle. Accordingly, the absorption coefficient for multiple reflections is $\alpha_{mr} = 1 - \rho_{mr}$. The total absorption, including the first absorption, was then calculated using $\alpha_{tot} = 1 - (1 - \alpha_{Fr}) * (1 - \alpha_{mr})$, where α_{Fr} is the Fresnel absorptivity taken from [6].

Supplementary Method 2

Experiment design

Thin walls were fabricated on the center of the substrate in standard keyhole printing conditions with a Gaussian beam at the focal point, focused on top of the powder bed with a measured beam diameter of 27.5 µm (see **Supplementary Figure 1a**). The keyhole regime was confirmed through ex-situ microstructural analysis and observation of the melt pool geometry. The laser process parameters of the build are reported in **Supplementary Table 1** – Process parameters for fabrication of the parts and for the subsequent re-melting (RM) passes. After the fabrication of the thin walls, as illustrated in **Supplementary Figure 2**, the build plate was moved upwards by the nominal height of the wall, and all the surrounding powders were removed to allow *operando* X-ray imaging through the entire thickness of the wall during laser processing of the top surface. Additionally, the top surface was ground slightly to remove the bulges formed at both ends of the wall as a result of laser acceleration and deceleration. Afterwards, single-line laser re-melting was carried out on the center line of the top surface of the wall, utilizing different laser process parameters and laser defocus values. In particular, converging (favoring keyhole formation) and diverging (stabilizing the conduction regime) defocus [3] with similar beam spot size and process parameters were employed to record the acoustic feedback from the laser-material interaction, to guarantee identical associated machine and environmental noises (e.g., noise transmitted from the laser at different nominal laser powers, scan head mirrors, etc.). The re-melting passes were repeated several times to improve the statistical significance of the measurements. The motivation behind the choice of wall thickness was twofold. Primarily, it had to be thin enough to provide adequate X-ray transmission, making high-speed X-ray imaging possible. Additionally, the effects of top and side surface roughness and geometrical inaccuracy become stronger for lower wall thicknesses [4], providing non-uniform heat dissipation and projected laser energy distribution during the re-melting process and leading to eventual changes in the melt pool geometry or even in the melting regime. The laser beam profile at each defocus value was measured using a CCD camera laser beam profiler (FBP-1KF, CINOGY Technologies, Germany) based on a multi-stage high-performance attenuator with a pixel size of 3.45 µm^2. The beam profile was displayed using a beam profiler software (RayCi64 Pro, CINOGY Technologies, Germany).

For non-Gaussian beam profiles, the beam diameter in three axes and the corresponding average value was measured at different intensity levels (20% - 80%) (see **Supplementary Figure 1b-f**). The theoretical evolution of the laser beam spot size at different defocus values was calculated based on the following equation (Eq.1).

$$\omega(z) = \omega_0\sqrt{1 + \left(\frac{\lambda(z-z_0)}{\pi w_0^2}\right)^2}, (1)$$

where ω_0 = 13.75 µm is the beam waist (i.e., the smallest radius of the Gaussian beam); z is the scanner position [mm]; z_0 = 0 mm is the scanner position for the laser beam to be focused on the surface of the build plate; λ = 1070 ± 10 nm is the laser wavelength.

Denomination	Nominal laser power (W)	Scanning velocity (mm/s)	Hatch distance (μm)	Layer thickness (μm)	Defocus distance (mm)	Laser defocus direction	Number of passes	Line Energy (J/m)
**Building parameters**	150	280	40	30	0	Focal point	n.d.	n.d.
**RM1**	200	40	-	-	1.7	Diverging	1	5000
**RM2**	186	40	-	-	1.7	Diverging	1	4650
**RM3**	200	30	-	-	2.1	Diverging	1	6667
**RM4**	365	100	-	-	2.1	Diverging	1	3650
**RM5**	447	150	-	-	2.1	Diverging	1	2980
**RM6**	186	40	-	-	-1.9	Converging	1	4650
**RM7**	186	40	-	-	-1.9	Converging	2	4650

Supplementary Table 1 *– Process parameters for fabrication of the parts and for the subsequent re-melting (RM) passes.*

Supplementary Method 3

Segmentation algorithm

In our study, we began by conducting exploratory data analysis (EDA) on the pre-processed signal spectrograms. As seen in **Figure 6** (of the main text), we observed that the majority of the signal energy was concentrated in the frequency range of 35–105 kHz when the process was in the keyhole regime. This observation prompted us to apply a digital filter to extract only the relevant frequency range. To improve the pipeline's expressivity, we applied a non-linear function point-wise to the filtered signal and smoothed it using a moving average. The selected non-linear function is PReLU [7], which facilitates the application of the successive smoothing filters by making the signal running average positive (the former is zero in the raw data due to the oscillatory nature of AE signals). This process is illustrated in **Supplementary Figure 3a**, where one of the pre-processed AE signals and the result of all the aforementioned operations are shown.

Additionally, we duplicated the filtering pipeline to produce two signals, each corresponding to a specific regime of interest. To do this, we initialized two filters: a band-pass filter with a passband frequency range of 35–105 kHz and a band-stop filter with the same stopband frequency. The impulse response duration was set to 1.25 ms, corresponding to 2501 data points at a 2 MHz sampling rate, to achieve at least 80 dB of attenuation (corresponding to the signals' mean SNR) for the band-pass (or band-stop) filter.

The implementation of two filtering branches offers several benefits to our approach. Firstly, it increases the robustness of the procedure as, even in the case of uncertain detection from one of the signals, the prediction can be supported by the second signal. Secondly, it creates a framework that can be easily expanded to detect multiple regimes by adding additional filtering branches. Lastly, it allows for generating predictions by applying the SoftMax function to the output of our filters. Specifically, the following SoftMax function is point-wise applied to the filtered signals:

$$\sigma_c(x_{n,i}) = \frac{e^{c(x_{n,i})}}{e^{c(x_{n,i})} + e^{k(x_{n,i})}}, \quad (3)$$

$$\sigma_k(x_{n,i}) = \frac{e^{k(x_{n,i})}}{e^{c(x_{n,i})} + e^{k(x_{n,i})}}, \quad (4)$$

where $x_{n,i}$ denotes the *i*-th data point of the *n*-th pre-processed AE signal x_n, $c(x_{n,i})$ and $k(x_{n,i})$ are the *i*-th data points of the *n*-th conduction and keyhole signals, respectively, and $\sigma_c(x_{n,i})$ ($\sigma_k(x_{n,i})$) is the prediction denoting conduction (or keyhole) detection for the *i*-th time step of x_n. As the name suggests, the SoftMax function finds the maximum between the two signals, which corresponds to our prediction; e.g., if the conduction signal ($c(x_{n,i})$) is higher than the keyhole one ($k(x_{n,i})$) at a specific time — which translates to $\sigma_c(x_{n,i})$ being close to 1 — we are predicting conduction at that time. Conversely, if $k(x_{n,i})$ is larger than $c(x_{n,i})$ — i.e., $\sigma_c(x_{n,i})$ is small, close to 0 — we are predicting the keyhole at the *i*-th time step of x_n. See **Supplementary Figure 4a** for the complete block diagram. Notice that by providing a prediction per data point, the complete pipeline results in a segmentation algorithm with the goal of dividing each AE signal into segments, each corresponding to a melting regime between conduction, stable keyhole, and unstable keyhole.

Our approach to segmenting the melting regimes for LPBF processes is built upon initial empirical observations of the available data, specifically the time-frequency analysis of the signals. Through this analysis, we were able to identify specific spectral characteristics that correspond to the conduction and keyhole regimes. However, we recognized that relying solely on these observations may not deliver the most optimal results for all available signals. Therefore, to optimize the discrimination of the regimes, we adopted a data-driven approach to improve the pipeline further.

This approach utilizes annotated data to fine-tune the pipeline, going beyond the initial time-frequency insights and applying a data-driven filter that allows one of the two output signals to be more intense when the corresponding regime is occurring. This fine-tuning allows for a more accurate and reliable segmentation of the regimes, which is crucial for real-time in-situ monitoring of the LPBF process.

Precisely, to guide the filter design optimization, we have used a variation over the cross-entropy loss, which ensures a low score when the pipeline segmentation matches the ground truth and a high one otherwise — e.g., when $\sigma_c(x_{n,i})$ is close to zero, even though $x_{n,i}$ should correspond to conduction mode. The ground truth in this context refers to the annotated data that provides the true labels — derived from the X-ray movies, see Section 2.1 — for each data point, indicating whether it corresponds to the conduction or keyhole regime. Specifically, the risk function (cross-entropy through time) is defined as follows:

$$R_D(\vartheta_c, \vartheta_k, x_n) = -\frac{1}{T_n}\sum_{i=1}^{T_n} \beta_k \gamma(x_{n,i}) \ln\left(\sigma_k(x_{n,i})\right) + \beta_c \left(1-\gamma(x_{n,i})\right) \ln\left(\sigma_c(x_{n,i})\right), \quad (5)$$

where T_n denotes the number of data points the signal x_n is made up of, $\gamma(x_{n,i})$ = 1 if $x_{n,i}$ corresponds to keyhole and $\gamma(x_{n,i})$ = 0 if $x_{n,i}$ corresponds to conduction mode according to the ground truth. The parameters β_k and β_c act as balancing coefficients between keyhole and conduction occurrences, which are not equally distributed, and their values are given by:

$$\beta_k = \frac{\sum_j I[GT_j=0] + I[GT_j=1]}{2\sum_j I[GT_j=1]}, \quad \beta_c = \frac{\sum_j I[GT_j=0] + I[GT_j=1]}{2\sum_j I[GT_j=0]}, \quad (6)$$

where I is an indicator function, whose value is 1 if the condition in the argument is true and 0 otherwise, and GT_j is the *j*-th element of the set containing all ground truth values (0 for conduction and 1 for keyhole) for all the available signals. Scaling the risk function by these two balancing coefficients reduces the penalty of wrong predictions for the most common regime while increasing it for the less frequent one.

Additionally, note that the risk R_D also depends on ϑ_c and ϑ_k, which represents the vectors comprising all the filters parameters (defining their impulse response), for both conduction (ϑ_c) and keyhole (ϑ_k) filtering branches.

Once the risk R_D is computed for all the available AE signals x_n using the initial parameters ϑ_c and ϑ_k, the latter can be updated as follows:

$$\vartheta_c \leftarrow \vartheta_c - \alpha \sum_{n=1}^{N} \nabla_{\vartheta_c} R_D(\vartheta_c, \vartheta_k, x_n), \quad (7)$$

$$\vartheta_k \leftarrow \vartheta_k - \alpha \sum_{n=1}^{N} \nabla_{\vartheta_k} R_D(\vartheta_c, \vartheta_k, x_n), \quad (8)$$

in which the gradients are calculated with respect to ϑ_c and ϑ_k, α is the so-called learning rate that controls the entity of the "step" taken towards the opposite gradient direction, and N denotes the number of signals used to optimize the filters' parameters. This technique is commonly referred to as gradient descent [8], and, in our case, the gradient calculations are performed with an automatic differentiation tool (Pytorch [9]).

Finally, repeating the steps denoted in Eqs. (7) and (8) using the value for the risk function obtained with the updated parameters ϑ_c and ϑ_k allows determining the parameters that minimize the risk function.

From binary to ternary

In the previous section, we introduced a regime segmentation technique based on the analysis of the acoustic emissions signals acquired during the Laser Powder Bed Fusion (LPBF) process. This approach relied on a filtering pipeline designed to extract specific frequency ranges from the raw data and a prediction model that utilized the SoftMax function to discriminate between the conduction and keyhole regimes. However, the flexibility of this technique suggests taking the optimization one step further by allowing for the discrimination of the unstable keyhole regime from the stable one.

This task was achieved through minor modifications to the previously described pipeline, which included the inclusion of one additional filtering branch and slight changes in the loss function so that it can handle a vectorial indicator function, i.e., making $\gamma(x_{n,i})$ one of the columns of the identity matrix of size K (the number of regimes we are predicting). The remaining required changes can be seen in **Supplementary Figure 4b**, where the complete pipeline for the segmentation of the three regimes mentioned above is presented. The procedure is detailed in **Supplementary Figure 3b**, where one of the pre-processed AE signals and the result of all the operations are presented. This extended capability is of extreme importance for LPBF processing monitoring, especially the detection of instabilities within the keyhole regime (where porosity formation occurs). Thanks to the high time resolution in these predictions, the location of the affected regions can be identified accurately, and healing measures can be employed to save processing time and resources.

Training procedure

The training procedure for the regime segmentation technique is divided into several steps. First, a leave-one-out cross-validation strategy is employed, where a single signal is left out from the training set and used as the test set. This process is repeated for all signals in the training set, providing a robust evaluation of the model performance.

We use a variation over the cross-entropy loss to guide the filter design optimization. The parameters of the pipeline are updated using the L-BFGS optimizer, an optimization algorithm that approximates the Newton Method using a limited memory of previous gradients. Additionally, we use a learning rate scheduler, which randomizes the learning rate if the risk does not decrease between one epoch and the following one. If the risk function does not decrease for 100 epochs, we apply a small amount of random noise to the signals to ensure that the pipeline does not get stuck in a suboptimal solution. The optimization process for every fold of the leave-one-out cross-validation is stopped according to the following criteria: when the minimum risk (a measure of the difference between the predicted and ground truth labels) is less than 0.1, or when the number of epochs is greater than 10'000, or when the "counter flat" reaches 100 and the minimum risk is less than 0.35. The "counter flat" is a value that is incremented

every time the risk does not decrease between one epoch and the following one, serving as a way to check if the optimizer has stopped making progress.

Supplementary Method 4

Materials

A gas-atomized 316L stainless steel (1.4404) powder (OC Oerlikon Corporation AG, Switzerland) with the chemical composition listed in **Supplementary Table 2** and a particle size distribution in the range of 15 to 45 µm was selected in this study. The morphology of the powder particles was observed as predominantly spherical, with the presence of occasional satellites.

Fe	Cr	Ni	Mo	Si	C	Other
**Balance**	17	12	2.5	2.3	0.03	≤ 0.5

Supplementary Table 2 *– Nominal chemical composition of the 316L stainless steel powder feedstock (wt%).*

Supplementary Method 5

LPBF apparatus

A miniaturized LPBF device designed at the Paul Scherrer Institute (PSI) [1] was chosen for this study. This setup mimics the commercial LPBF process and can be incorporated into synchrotron beamlines for *in-situ* X-ray measurements simultaneously. Its relatively small dimensions (height: 520 mm, width and depth: 280 mm and 260 mm, respectively) and lightweight (25 kg) make it easily transportable to synchrotron beamlines, while the presence of two glassy carbon windows allows the X-ray beam to access the powder bed from the rear window and the transmitted X-ray beam to reach the detector placed outside of the chamber through the front window. **Supplementary Figure 2** displays the main components of the mini-LPBF device. Its printing chamber contains a 12 x 12 mm^2 build plate and a hopper-based recoating system equipped with several doctor blades. Prior to each recoating, the build plate is moved down by a distance corresponding to the layer thickness. The powder is then delivered by gravity through the hopper, with the excessive powder scrapped away via the doctor blades. During and before the operation, the chamber is flushed with high-purity Argon gas (99.996%) from several inlets, including a small cavity designed in the recoating system, ensuring the removal of by-products from the laser-material interaction zone. The oxygen level is monitored continuously to ensure its stability below 2000 ppm. A more detailed description of the device can be found in [1,2].

A dual-mode fiber laser (redPOWER, SPI Lasers Ltd, UK) with a maximum laser power of 500 W operating at a wavelength of 1070 ± 10 nm with a beam quality factor of $M^2 < 1.1$ was used. Both laser and the scanning head are piloted via an SP-ICE-3 board and WeldMARK software (Raylase GmbH, Germany). The laser beam is collimated as a parallel Gaussian beam into a 2-axis deflection-scanning unit (SuperScan III, Raylase GmbH, Germany) with an 8 mm input aperture. Two HR-coated fused silica mirror galvanometers allow scanning of the laser beam over the powder bed. The beam is focused through an *F*-Theta lens (Sill Optics, Germany) with a 163 mm focal length resulting in a focused beam with a spot size of 27.5 µm in diameter at $1/e^2$ and a depth of field of about 1 mm. The laser was utilized in pulsed mode with a pulse repetition rate of 250 kHz and a nominal laser pulse duration of 2 µs.

Supplementary Method 6

Signal pre-processing

In our experiments, the acoustic data acquisition was triggered manually shortly before the start of the laser irradiation. However, due to the high signal-to-noise ratio (SNR), the beginning and the end of laser processing were clearly detectable in the XARION raw acoustic data, allowing for the removal of the signal sections corresponding to the laser being off by simple thresholding. This operation is hereafter referred to as preliminary segmentation. The so-processed signals are then normalized using the RobustScaler algorithm according to the following formula:

$$x_{n,i} = \frac{r_{n,i} - P_1(r_n)}{P_{99}(r_n) - P_1(r_n)}, \quad (2)$$

where $r_{n,i}$ and $x_{n,i}$ denote the *i*-th data point of the *n*-th raw AE signal r_n and the *n*-th pre-processed AE signal x_n, respectively, and $P_{99}(r_n)$ and $P_1(r_n)$ are the 99th and the 1st percentiles of r_n. This operation is more insensitive to outliers compared to *min-max* normalization — where $P_1(r_n)$ is replaced by $\min(r_n)$ and $P_{99}(r_n)$ by $\max(r_n)$ — making the whole process less dependent on the signal amplitude and the distance from the process zone to the microphone.

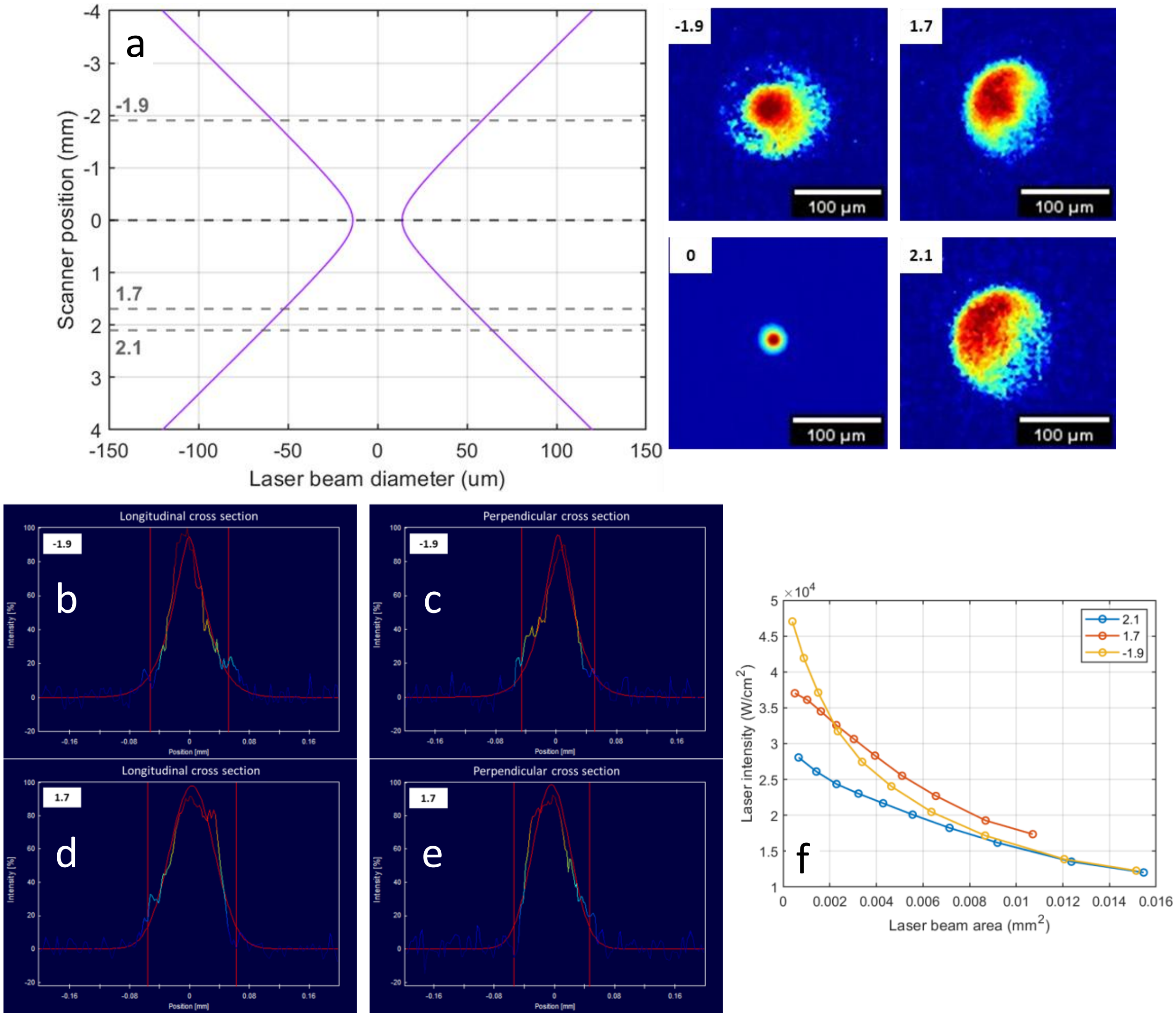

Supplementary Figure 1 *–* ***Analysis of the laser beam shapes.*** *(a) Theoretical Gaussian laser beam profile alongside the z-axis calculated via Eq.1 and the corresponding measured beam profiles via CCD camera laser beam profiler. Due to a small misalignment of the laser beam through the optical chain, a deviation from the Gaussian profile is observed in both defocus directions. Cross-sectional laser beam profiles alongside (longitudinal) and perpendicular to the moving direction of the laser for (b and c) negative defocus (-1.9) and (d and e) positive defocus (1.7). The Gaussian fit provides a reference for the deviations. The cross-sectional line passes through the centroid of the beams in all cases. (f) Average laser intensities per area for different beam shapes using 186 W nominal laser power (RM1, RM2, RM6 and RM7).*

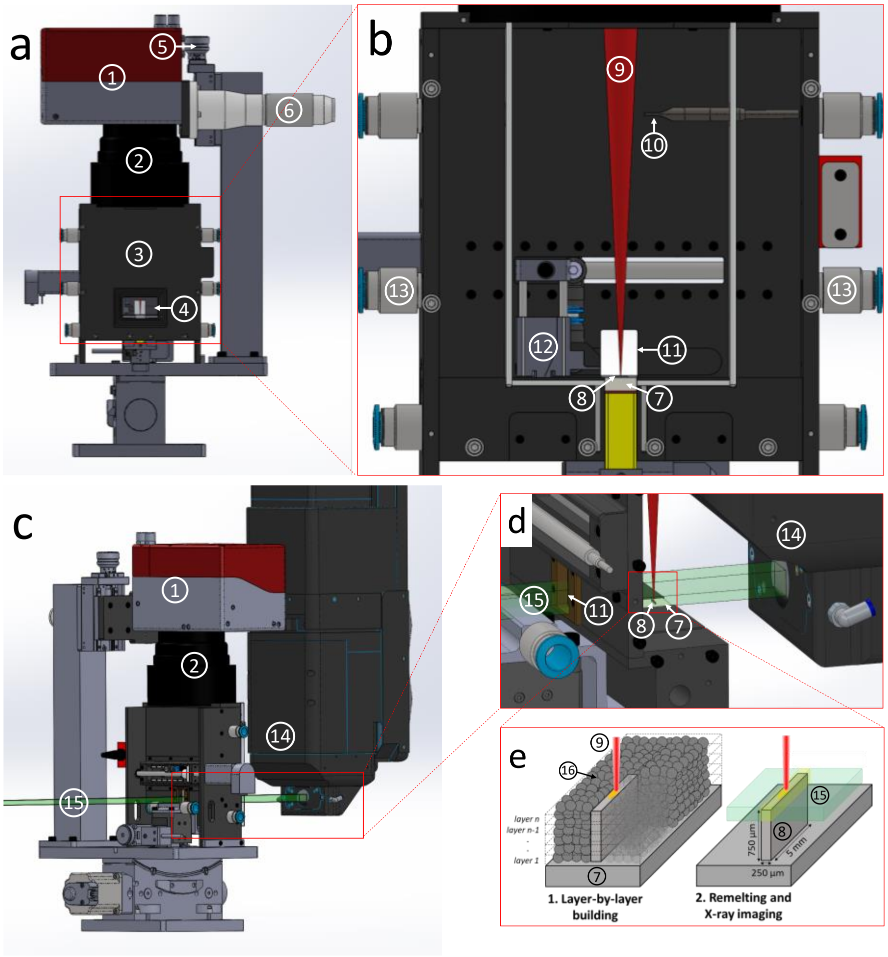

***Supplementary Figure 2** – **Front view of the miniaturized LPBF device.** (a) with and (b) without the front door; schematic view of operando x-ray radiography setup at the synchrotron beamline (c) and its magnification (d); (e) thin wall geometry in presence and absence of powder bed. 1) scanning head, 2) F-theta lens, 3) front door, 4) X-ray exit window, 5) vertical stage to change focal length, 6) laser collimator, 7) build plate, 8) sample, 9) laser beam, 10) AE microphone, 11) X-ray entrance window, 12) powder hopper and recoater, 13) gas inlet and outlet, 14) microscope, 15) polychromatic X-ray beam, 16) powder bed.*

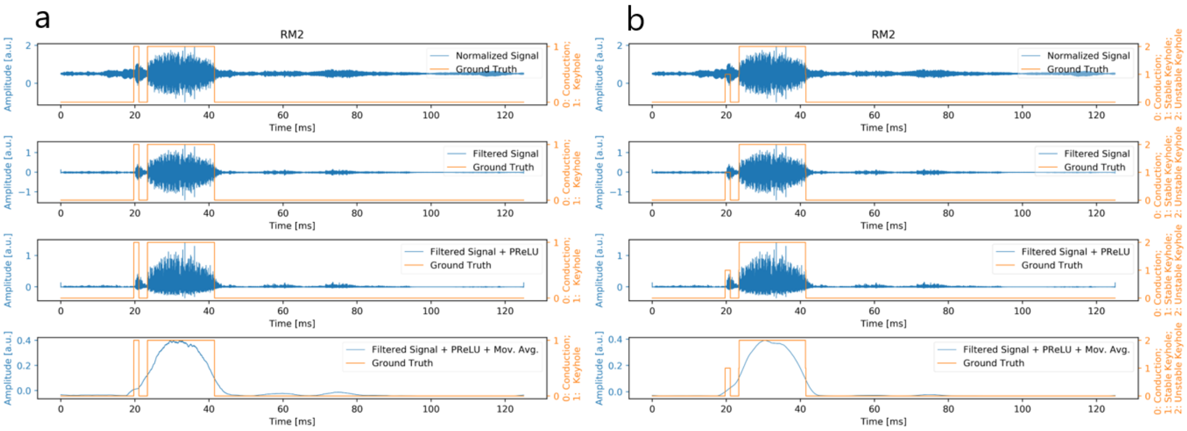

***Supplementary Figure 3** – **Pre-processing of the AE signals.** (a) In blue, the time evolution of one signal (RM2) after being processed by each element of the keyhole filtering branch of the binary processing pipeline before any optimization. In orange, the time evolution of the ground truth, which has a value of 1 (0) when the keyhole (conduction) regime is occurring. The shown signal is obtained after pre-processing (including preliminary segmentation and normalization) and thus contains data acquired only during laser processing. (b) In blue, the time evolution of one signal (RM2) after being processed by each element of the stable keyhole filtering branch of the ternary processing pipeline before any optimization. In orange, the time evolution of the ground truth, which has a value of 0, 1, and 2 when the conduction regime, stable keyhole, and unstable keyhole are occurring, respectively. The shown signal is obtained after pre-processing (including preliminary segmentation and normalization) and thus contains data acquired only during laser processing.*

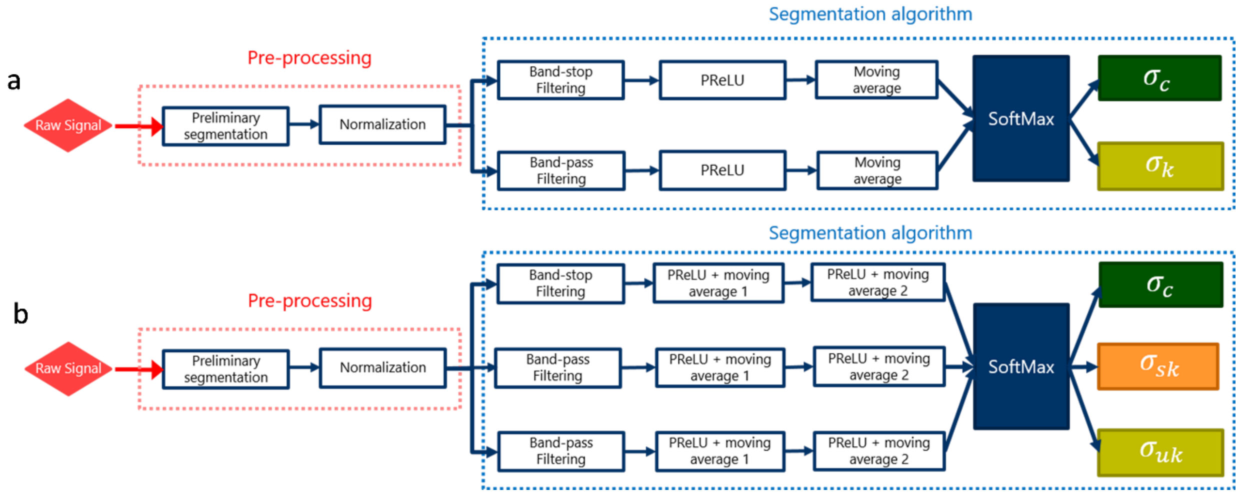

***Supplementary Figure 4** – **Block diagrams illustrating the entire processing pipelines for binary and ternary segmentation challenges.** (a) Block diagram of the complete processing pipeline for the binary segmentation problem. The pre-processing stage includes preliminary segmentation — to remove the signal sections acquired before and after laser processing — and normalization — to be less sensitive to the signal amplitude and the distance from the sample to the microphone. The following segmentation stage includes two filtering branches — each made up of an actual filter, a non-linear function, a moving average, and a SoftMax function — and produces two signals with the same duration (and the same number of data points) as the original raw data. For every time step, the prediction on which processing regime is occurring can be derived by checking which of the two output signals — σ_c (for conduction) and σ_k (for keyhole) — is closer to 1. (b) Block diagram of the complete processing pipeline for the ternary segmentation problem. The pre-processing stage includes the same stages used for the binary pipeline. The following segmentation stage includes three filtering branches — each made up of an actual filter, a non-linear function followed by a moving average, a second non-linear function followed by a moving average, and a SoftMax function — and produces three signals with the same duration (and the same number of data points) as the original raw data. Same as for the binary case, for every time step, the prediction on which processing regime is occurring can be derived by checking which of the three output signals — σ_c (for conduction), σ_{sk} (for stable keyhole), and σ_{uk} (for unstable keyhole) — is closer to 1.*

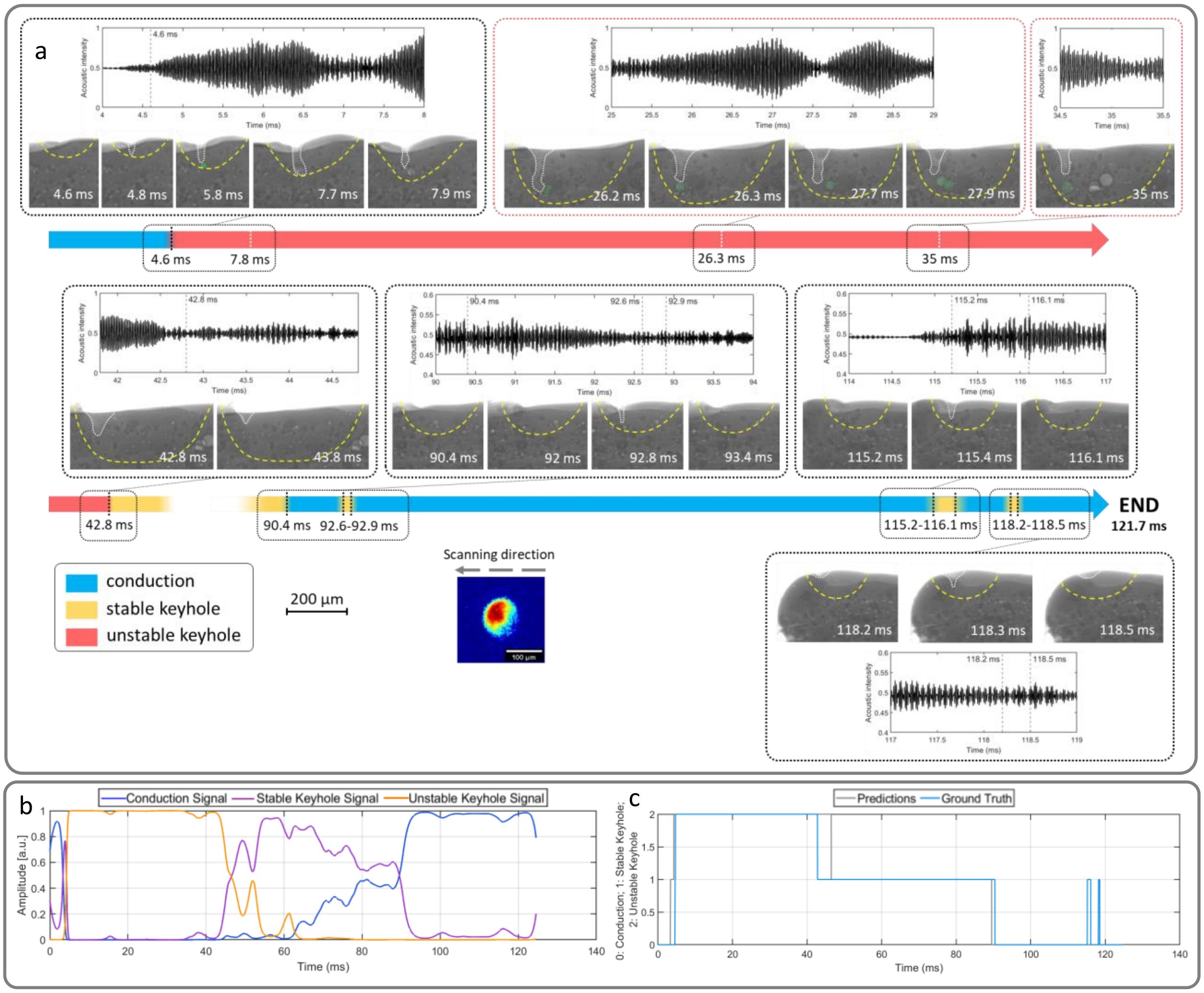

Supplementary Figure 5 – Stochastic regime instabilities and subsequent transition from conduction to stable and unstable keyhole regimes in RM1 condition*. (a) RM1 melt pool morphology variations under constant laser process parameters in a single scanning vector of 316L stainless steel (c.f. Supplementary movie 1). The normalized and filtered acoustic signal for each time frame is illustrated for each image set with event annotations represented by vertical lines. The boundaries of solid-liquid (melt pool) and gas-liquid (depression zone) are illustrated by yellow and white dashed lines, respectively. The time bar depicts the regime changes from conduction to unstable keyhole (Ev1, t = 4.6 ms), unstable keyhole to stable keyhole (Ev2, t = 42.8 ms), stable keyhole to conduction (Ev3, t = 90.4 ms), and conduction to stable keyhole and back to conduction taking place repetitively at (Ev4, t = 92.6 – 92.9 ms), (Ev5, t = 115.2 – 116.1 ms), and (Ev6, t = 118.2 – 118.5 ms) respectively. The resultant pores in the unstable keyhole regime in the vicinity of each timeline are encircled by dashed green lines. The scale bar for all the images is shown in the bottom left corner. The laser intensity profile (1.7, c.f. Supplementary Figure 1) and the corresponding scanning direction are presented at the center bottom of the figure. (b) Time evolution of the output of the segmentation algorithm (after SoftMax normalization) obtained for RM1 signal for ternary segmentation problem. (c) time evolution of the ground truth (blue) alongside the predictions obtained (gray). As can be seen, the optimized filters can discern between the regimes by producing signals with a high value when the corresponding regime is occurring and a low one when the other regime is detected.*

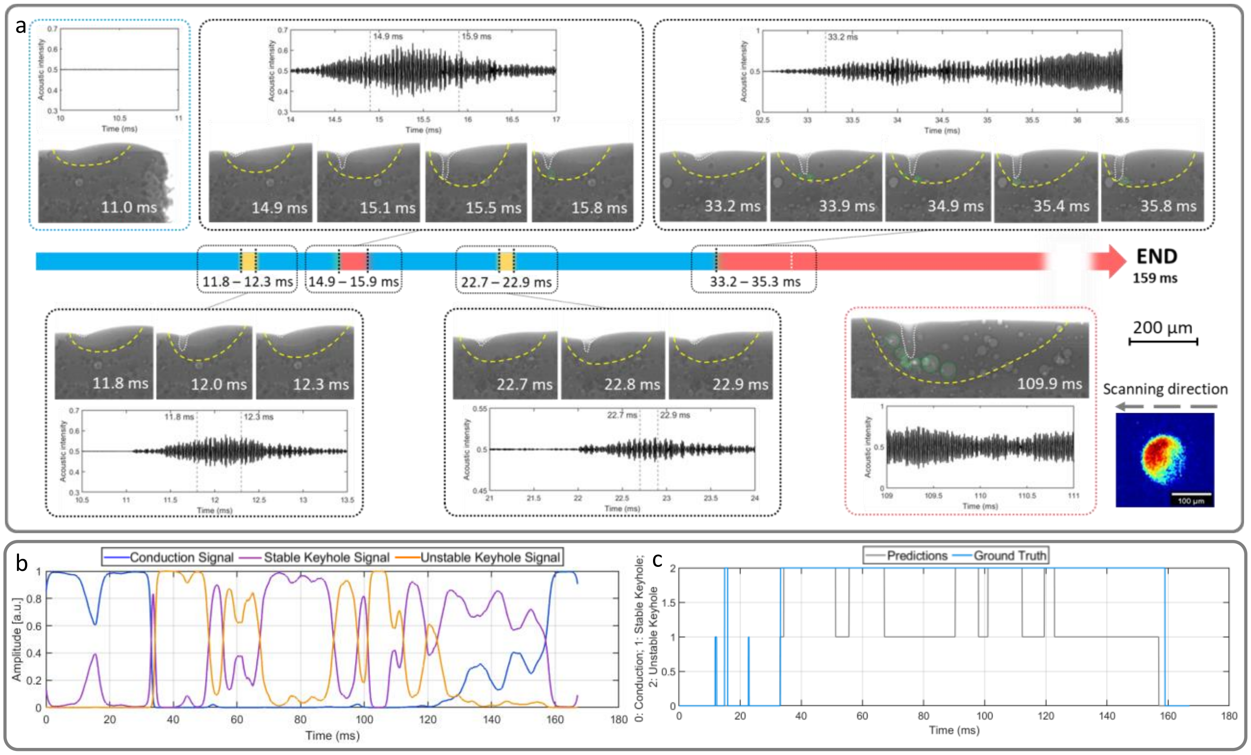

***Supplementary Figure 6** – **Stochastic regime instabilities and subsequent transition from conduction to stable and unstable keyhole regimes in RM3 condition**. (a) RM3 melt pool morphology variations under constant laser process parameters in a single scanning vector of 316L stainless steel (c.f. Supplementary movie 3). The normalized and filtered acoustic signal for each time frame is illustrated for each image set with event annotations represented by vertical lines. The boundaries of solid-liquid (melt pool) and gas-liquid (depression zone) are illustrated by yellow and white dashed lines, respectively. The time bar depicts regime changes from conduction to stable keyhole and back to conduction (Ev1, t = 11.8 – 12.3 ms), conduction to unstable keyhole and back to conduction (Ev2, t = 14.9 – 15.9 ms), conduction to stable keyhole and back to conduction (Ev3, t = 22.7 – 22.9 ms), and conduction to unstable keyhole (Ev4, t = 33.2 ms). The resultant pores in the unstable keyhole regime in the vicinity of each timeline are encircled by dashed green lines. The scale bar for all the images is shown in the top right corner. The laser intensity profile (2.1, c.f. Supplementary Figure 1) and the corresponding scanning direction are presented in the bottom right corner. (b) Time evolution of the output of the segmentation algorithm (after SoftMax normalization) obtained for RM3 signal for ternary segmentation problem. (c) time evolution of the ground truth (blue) alongside the predictions obtained (gray). As can be seen, the optimized filters can discern between the regimes by producing signals with a high value when the corresponding regime is occurring and a low one when the other regime is detected.*

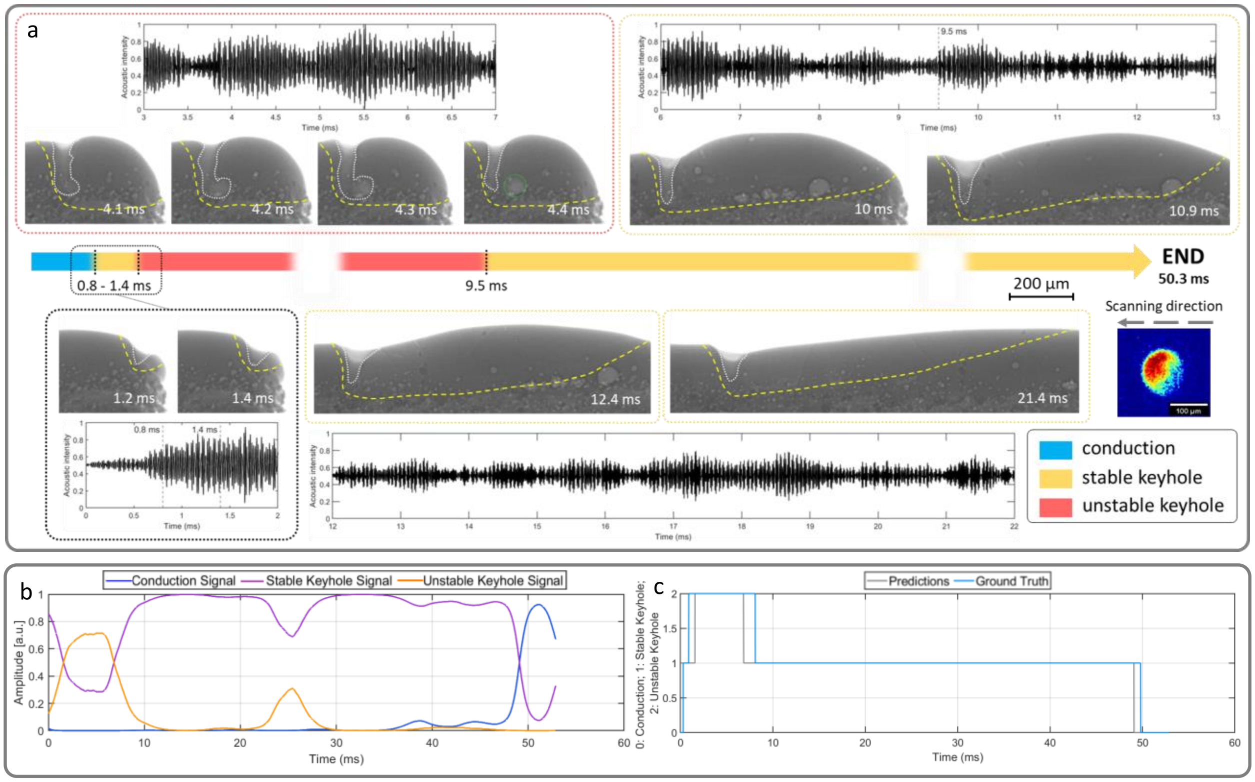

***Supplementary Figure 7** – **Stochastic regime instabilities and subsequent transition from conduction to stable and unstable keyhole regimes in RM4 condition**. (a) RM4 melt pool morphology variations under constant laser process parameters in a single scanning vector of 316L stainless steel (c.f. Supplementary movie 4). The normalized and filtered acoustic signal for each time frame is illustrated for each image set with event annotations represented by vertical lines. The boundaries of solid-liquid (melt pool) and gas-liquid (depression zone) are illustrated by yellow and white dashed lines, respectively. The time bar depicts the regime changes from conduction to stable keyhole (Ev1, t = 0.8 ms), stable to unstable keyhole (Ev2, t = 1.4 ms), and unstable to stable keyhole (Ev3, t = 9.5 ms). The resultant pores in the unstable keyhole regime in the vicinity of each timeline are encircled by dashed green lines. The scale bar for all the images is shown in the top right corner. The laser intensity profile (2.1, c.f. Supplementary Figure 1) and the corresponding scanning direction are presented in the bottom right corner. (b) Time evolution of the output of the segmentation algorithm (after SoftMax normalization) obtained for RM4 signal for ternary segmentation problem. (c) time evolution of the ground truth (blue) alongside the predictions obtained (gray). As can be seen, the optimized filters can discern between the regimes by producing signals with a high value when the corresponding regime is occurring and a low one when the other regime is detected.*

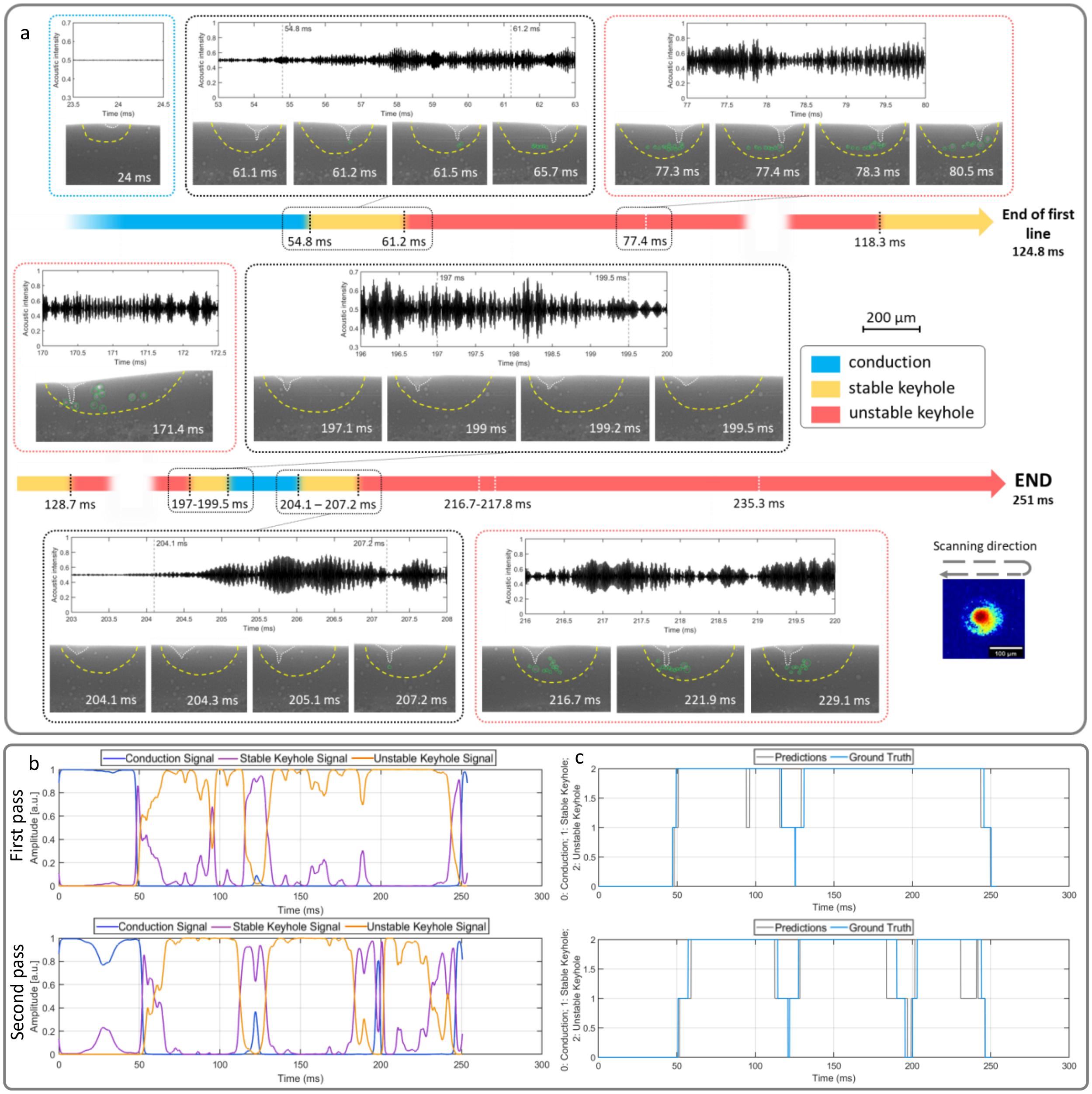

***Supplementary Figure 8** – **Stochastic regime instabilities and subsequent transition from conduction to stable and unstable keyhole regimes in RM7 condition**. (a) RM7 melt pool morphology variations under constant laser process parameters in a single scanning vector of 316L stainless steel (c.f. Supplementary movie 6). The normalized and filtered acoustic signal for each time frame is illustrated for each image set with event annotations represented by vertical lines. The boundaries of solid-liquid (melt pool) and gas-liquid (depression zone) are illustrated by yellow and white dashed lines, respectively. The time bar depicts the regime changes from conduction to stable keyhole (Ev1, t = 54.8 ms), stable to unstable keyhole (Ev2, t = 61.2 ms), unstable to stable keyhole (Ev3, t = 118.3 ms), end of the first line scan (t = 124.8 ms), stable to unstable keyhole (Ev4, t = 128.7 ms), unstable to stable keyhole (Ev5, t = 197 ms), stable to conduction and back to stable keyhole (Ev6, t = 199.5 – 204.1 ms), stable to unstable keyhole (Ev7, t = 207.2 ms). The resultant pores in the unstable keyhole regime in the vicinity of each timeline are encircled by dashed green lines. The scale bar for all the images is shown in the top right corner. The laser intensity profile (-1.9, c.f. Supplementary Figure 1) and the corresponding scanning direction are presented in the bottom right corner. (b) Time evolution of the output of the segmentation algorithm (after SoftMax normalization) obtained for RM7 signal for ternary segmentation problem. (c) time evolution of the ground truth (blue) alongside the predictions obtained (gray). As can be seen, the optimized filters can discern between the regimes by producing signals with a high value when the corresponding regime is occurring and a low one when the other regime is detected.*

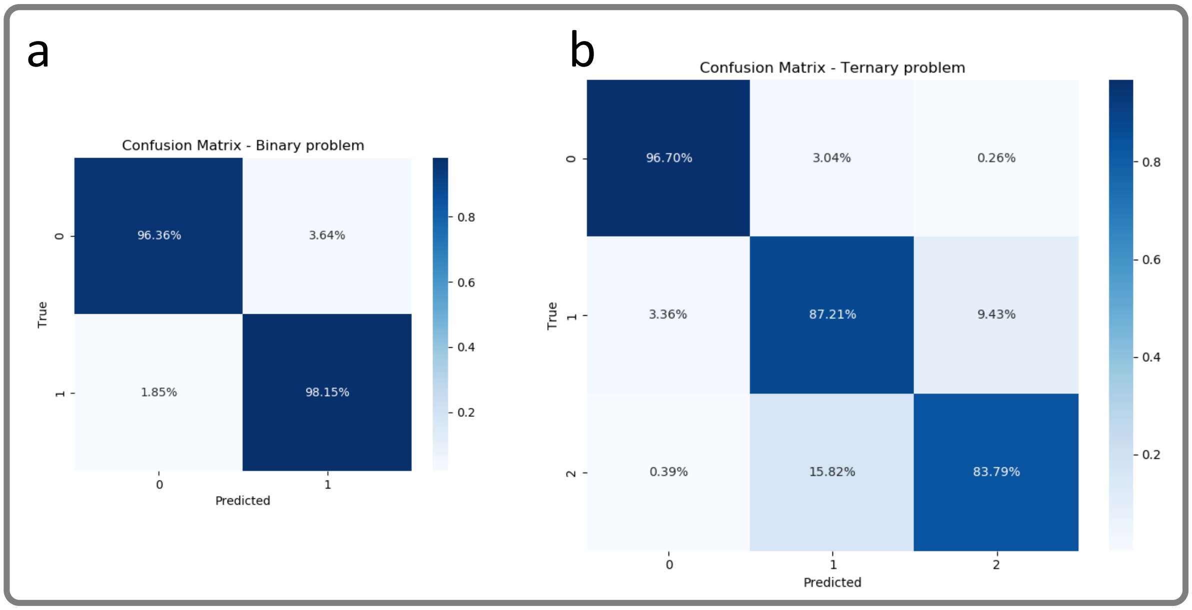

***Supplementary Figure 9** – **Heatmaps depicting the confusion matrices for both binary and ternary classification problems.** (a) heatmap of the confusion matrix for the binary classification problem, where 0 represents conduction, and 1 represents keyhole. (b) heatmap of the confusion matrix for the ternary classification problem, where 0 represents conduction, 1 represents stable keyhole, and 2 represents unstable keyhole. The matrices show the percentage of correct and incorrect predictions made by the algorithm, with the rows representing the true values and the columns representing the predicted values. The color scale indicates the percentage of the correct prediction, with darker shades indicating higher percentages.*

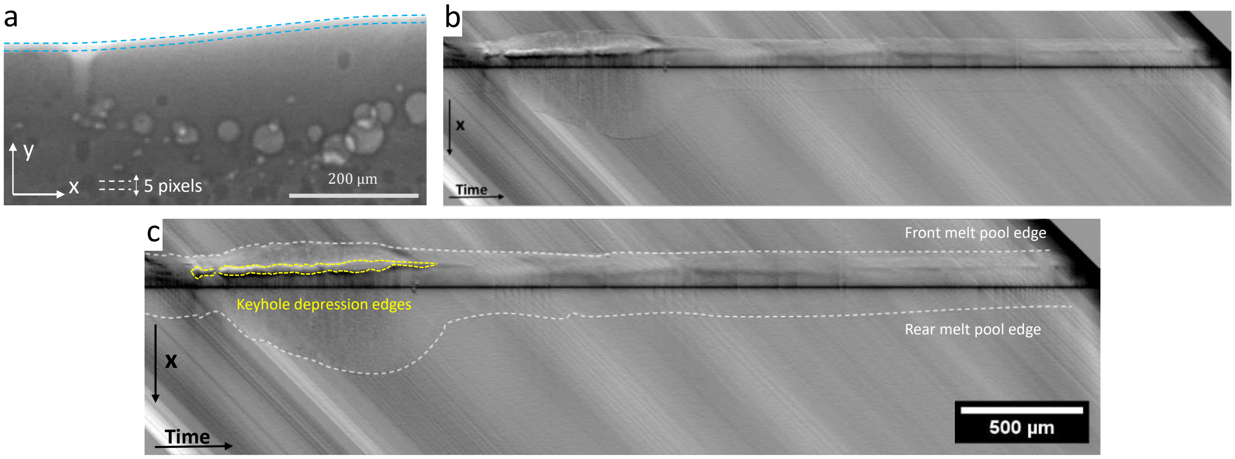

***Supplementary Figure 10** – **Detection of the melt pool and keyhole depression edges at the melt pool surface. (a)** Single frame, where, the top blue dashed line represents the top surface and the one below is placed within 5 pixels from it. The averaged 5 pixels throughout all y-pixel columns are assembled for each time frame creating a vertical array of pixels. **(b)** The 2D image consisted of assembled vertical arrays through time with the horizontal axis being time and the vertical axis being the vertical array of pixels from each timestep according to (a). **(c)** Annotated melt pool (white dashed lines) and keyhole depression edges (yellow dashed lines).*

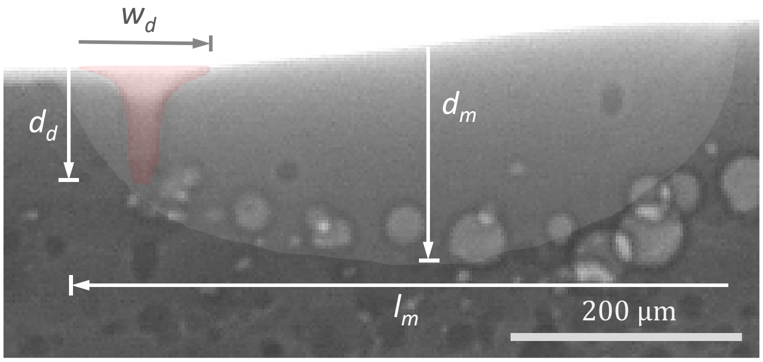

***Supplementary Figure 11** – **Measurement of the melt pool and depression zone dimensions.** The light area is the melt pool, and the red-shaded area corresponds to the depression zone (keyhole). The output of the image analysis and annotation are melt pool length (l_m), melt pool depth (d_m), depression width (w_d) and depression depth (d_d).*

Supplementary References

1. Hocine, S. *et al.* Operando X-ray diffraction during laser 3D printing. *Mater. Today* **34**, 30–40 (2020).
2. Hocine, S. *et al.* A miniaturized selective laser melting device for operando X-ray diffraction studies. *Addit. Manuf.* **34**, 101194 (2020).
3. Metelkova, J. *et al.* On the influence of laser defocusing in Selective Laser Melting of 316L. *Addit. Manuf.* **23**, 161–169 (2018).
4. Nasab, M. H., Gastaldi, D., Lecis, N. F. & Vedani, M. On morphological surface features of the parts printed by selective laser melting (SLM). *Addit. Manuf.* **24**, 373–377 (2018).
5. Kaplan, A. A model of deep penetration laser welding based on calculation of the keyhole profile. *J. Phys. D. Appl. Phys.* **27**, 1805–1814 (1994).
6. Huang, Y. *et al.* Keyhole fluctuation and pore formation mechanisms during laser powder bed fusion additive manufacturing. *Nat. Commun.* **13**, 1170 (2022).
7. He, K., Zhang, X., Ren, S. & Sun, J. Delving Deep into Rectifiers: Surpassing Human-Level Performance on ImageNet Classification. in *2015 IEEE International Conference on Computer Vision (ICCV)* vol. 2015 Inter 1026–1034 (IEEE, 2015).
8. Amari, S. ichi. Backpropagation and stochastic gradient descent method. *Neurocomputing* **5**, 185–196 (1993).
9. Paszke, A. *et al.* PyTorch: An imperative style, high-performance deep learning library. in *Advances in Neural Information Processing Systems* vol. 32 (Curran Associates Inc., 2019).
